# Coronavirus in cat flea: findings and questions regarding COVID-19

**DOI:** 10.1186/s13071-020-04292-y

**Published:** 2020-08-10

**Authors:** Margarita Villar, Isabel G. Fernández de Mera, Sara Artigas-Jerónimo, Marinela Contreras, Christian Gortázar, José de la Fuente

**Affiliations:** 1 SaBio. Instituto de Investigación en Recursos Cinegéticos IREC-CSIC-UCLM-JCCM, Ronda de Toledo s/n, 13005 Ciudad Real, Spain; 2 Biochemistry Section, Faculty of Science and Chemical Technologies, and Regional Centre for Biomedical Research (CRIB), University of Castilla-La Mancha, 13071 Ciudad Real, Spain; 3 Interdisciplinary Laboratory of Clinical Analysis, Interlab-UMU, Regional Campus of International Excellence Campus Mare Nostrum, University of Murcia, Espinardo, 30100 Murcia, Spain; 4 Department of Veterinary Pathobiology, Center for Veterinary Health Sciences, Oklahoma State University, Stillwater, OK 74078 USA

**Keywords:** Coronavirus, COVID-19, Arthropod, Flea, Cat, Proteomics, ACE

## Abstract

The coronavirus disease 19 (COVID-19) pandemic caused by severe acute respiratory syndrome coronavirus 2 (SARS-CoV-2) has affected millions of people worldwide. Recent evidence raised the question about the possibility that cats may be a domestic host for SARS-CoV-2 with unknown implications in disease dissemination. Based on the fact that the domestic cat flea, *Ctenocephalides felis*, are abundant ectoparasites infesting humans, companion animals and wildlife and that coronavirus-like agents have been identified in the ectoparasite tick vector, *Ixodes uriae* of seabirds, herein we considered the presence of coronaviruses in general and SARS-CoV-2 in particular in *C. felis*. We identified coronavirus-derived and cell receptor angiotensin-converting enzyme RNA/proteins in *C. felis*. Although current evidence suggests that pets are probably dead-end-hosts with small risk of transmission to humans, our results suggested that cat flea may act as biological and/or mechanical vectors of SARS-CoV. Although preliminary, these results indicate a possibility of ectoparasites acting as reservoirs and vectors of SARS-CoV and related beta-coronavirus although with little disease risk due to systemic transmission route, low viremia, virus attenuation or other unknown factors. These results support the need to further study the role of animal SARS-CoV-2 hosts and their ectoparasite vectors in COVID-19 disease spread.
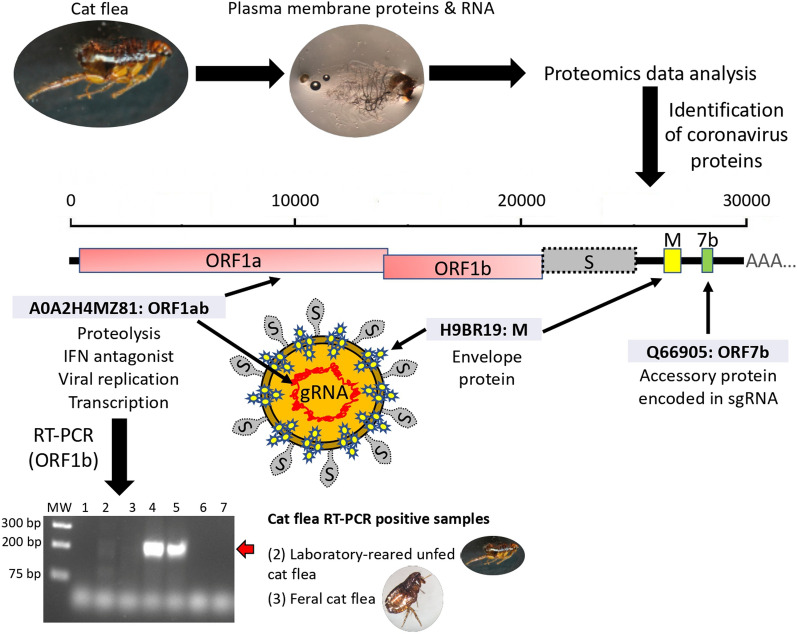

Letter to the Editor

The domestic cat flea, *Ctenocephalides felis* (Bouché, 1835) (Siphonaptera: Pulicidae), is an abundant ectoparasite infesting humans, companion animals and wildlife worldwide [1]. The disease risks associated with cat fleas include direct damage to the skin, discomfort, nuisance, allergic reactions, anemia, and transmission of pathogens such as *Yersinia pestis* (plague), *Rickettsia typhi* (murine typhus), *Rickettsia felis* (murine typhus-like illness), *Bartonella* spp. (cat-scratch disease) and calicivirus (feline gastroenteritis) that are of public health importance [1–3]. Fleas are also competent intermediate hosts of the tapeworm *Dipylidium caninum* (pulicosis) and the filarial nematode *Acanthocheilonema reconditum* (subcutaneous infection in animals and ocular disease in humans) [1]. *Bartonella vinsonii arupensis*, *Babesia microti*, and a *Rickettsia felis*-like bacterium have also been identified in flea samples (*Orchopeas leucopus*) collected from small mammals in Pennsylvania [4].

Coronaviruses (order Nidovirales, family Coronaviridae, subfamily Coronavirinae) are well known as infectious pathogens of humans and animals worldwide [5]. The first described coronavirus was isolated from chickens in 1937 and the model coronavirus is the mouse hepatitis virus (MHV) [6]. Many coronaviruses originate in bats and then through zoonotic transmission adapt their receptor-binding mechanisms to infect other animal species and humans [7]. Coronaviruses such as infectious bronchitis virus (IBV) and bovine coronavirus (BCoV) also affect domesticated animals and in particular chicken and cattle, respectively [6]. Furthermore, these viruses are known to cross the interspecies barrier and cause diseases such as severe acute respiratory syndrome and Middle East respiratory syndrome in domestic and wild ruminants [6].

The coronavirus disease 19 (COVID-19), a pandemic caused by severe acute respiratory syndrome coronavirus 2 (SARS-CoV-2), has affected millions of people worldwide [8]. SARS-CoV-2 infect human host cells by binding to the angiotensin-converting enzyme 2 (ACE2) receptor [9]. The exact origin of the SARS-CoV-2 has not been demonstrated but based on the proposed host for SARS-CoV causing the 2002–2003 pandemic, it is possible that the virus comes from bats such as Chinese horseshoe bats [8]. However, other animal species could act as intermediate animal hosts of the virus. Recently, a cat was reported infected with SARS-CoV-2 and developed both respiratory and enteric symptoms [10]. To identify potential animal host species, recent investigations showed that while SARS-CoV-2 replicates poorly in dogs, pigs, chicken and ducks, as other SARS viruses it efficiently replicates in ferrets and cats [11]. More recently, two cats and their owners tested positive for SARS-CoV-2 and both animals showed respiratory signs [10]. This evidence raised the question about the possibility that cats may be a domestic host for SARS-CoV-2 with unknown implications in disease dissemination.

To contribute addressing this question, herein we reused proteomics data in combination with RT-PCR to characterize the presence of coronaviruses in general and SARS-CoV-2 in particular in the domestic cat flea, an ectoparasite vector of *Yersinia*, *Rickettsia* and *Bartonella* spp. infecting humans and cats [1–3] (Fig. 1). Two previous findings are particularly relevant to this study, feline coronaviruses (FCoV) cause the usually fatal feline peritonitis (FIP) in cats [12], and coronavirus-like agents have been identified in the ectoparasite tick vector, *Ixodes uriae* of seabirds [13].

**Fig. 1:**
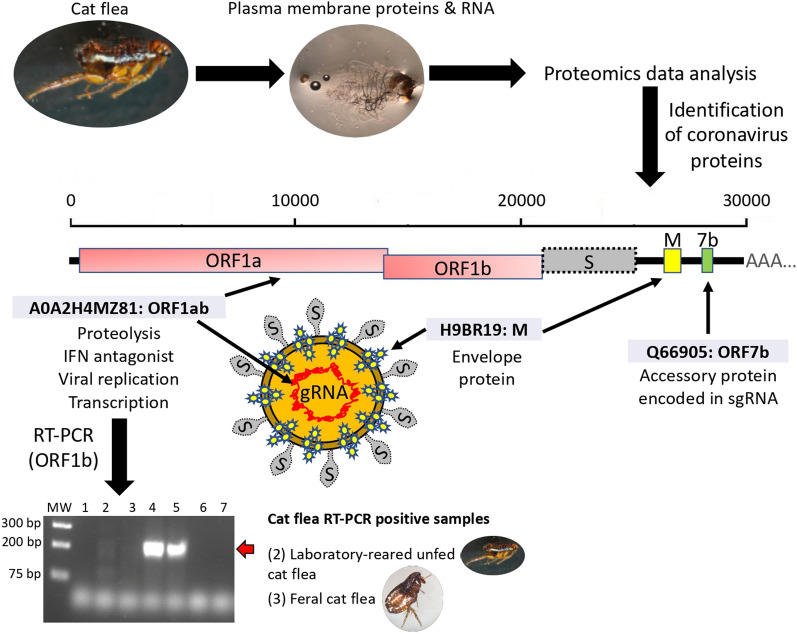
Experimental design and identification of coronavirus-derived RNA and proteins in cat flea. Representative images of a laboratory-reared domestic cat flea and tissues used for extraction of RNA and plasma membrane proteins for RT-PCR and proteomics analysis. Schematic representation of the coronavirus genome organization and virion structure based on SARS-CoV-2 [15]. Coronavirus proteins identified by proteomics analysis included ORF1a, ORF1b, protein M and protein 7b. Genomic RNA (gRNA) serves as mRNA for ORF1a and ORF1b. Other major subgenomic RNAs (sgRNAs) are produced to encode for envelope (e.g. protein M) and accessory (e.g. protein 7b) proteins in addition to the gRNA. Real-time RT-PCR targeting ORF1b identified in coronavirus-derived RNA in laboratory-reared unfed cat flea (sample 2) and feral cat flea (sample 3). MW, molecular weight O’GeneRuler 1 kb Plus DNA Ladder (Thermo Fisher Scientific); sample 1, laboratory-reared unfed cat flea, samples 4 and 5, positive controls (Table 1); samples 6 and 7, nuclease-free water negative controls

Laboratory-reared domestic cat fleas (European strain) were maintained by feeding on adult mixed breed shorthair cats [3]. Data were obtained from the previously reported proteomics analysis of plasma membrane proteins from mixed female and male (sex ratio approximately 50:50) unfed adult domestic cat fleas of similar age [3]. Pools of 12–20 unfed/partially fed fleas were collected from two feral cats sampled in Ciudad Real, Spain, during May-June 2020. Fleas were identified as *C. felis* according to morphological characters described by Linardi and Santos [14]. Methods are described in Fig. 2 and Table 1 legends.

**Fig. 2:**
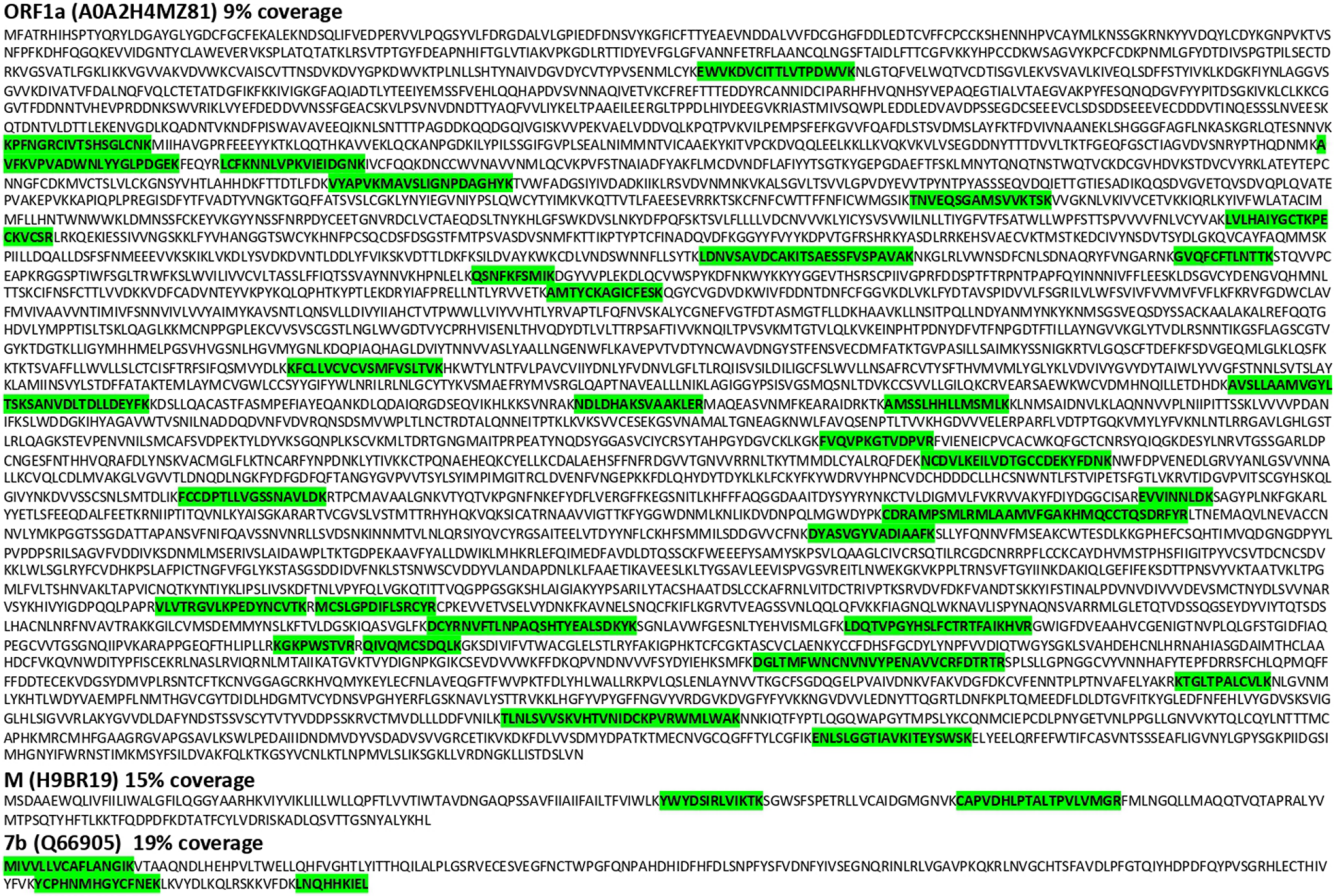
Coronavirus-derived sequences with the peptides identified by proteomics analysis and protein coverage. Proteins were analyzed by reverse phase (RP)-liquid chromatography (LC)-mass spectrometry (MS)/MS (RP-LC-MS/MS) using an Easy-nLC II system coupled to an ion trap LCQ Fleet mass spectrometer (Thermo Fisher Scientific) as previously reported [3]. For this study, MS/MS raw files were searched again against a compiled database containing all proteins from Felidae, coronavirus, *Drosophila* and Ctenocephalidae (78265, 30637, 342897 and 475 Uniprot entries in May 2020, respectively). Amino acid sequences of proteins corresponding to the Uniprot (https://www.uniprot.org) entries shown in parenthesis. The sequences of the peptides identified by proteomics analysis are highlighted in green and the sum of their amino acids reflect protein coverage when compared to the sequence of the identified proteins

**Table 1:** RT-PCR targets, oligonucleotide primers and results

RT-PCR target	Primers: 5′–3′ sequences (amplicon size)	Results (flea sample: Ct)
Coronavirus generic group ORF1b	11-FW: TGATGATGSNGTTGTNTGYTAYAA 13-RV: GCATWGTRTGYTGNGARCARAATTC (179 bp)	Fed: na Unfed: 35.24 Feral cat 1: 36.16 Feral cat 2: na
SARS-CoV-2, RdRp-IP2	nCoV_IP2-12669Fw: ATGAGCTTAGTCCTGTTG nCoV_IP2-12759Rv: CTCCCTTTGTTGTGTTGT nCoV_IP2-12696b: AGATGTCTTGTGCTGCCGGTA [5′]Hex [3′]BHQ-1 (108 bp)	Fed: na Unfed: na Feral cat 1: na Feral cat 2: na
SARS-CoV-2, RdRp-IP4	nCoV_IP4-14059Fw: GGTAACTGGTATGATTTCG nCoV_IP4-14146Rv: CTGGTCAAGGTTAATATAGG nCoV_IP4-14084: TCATACAAACCACGCCAGG [5′]Fam [3′]BHQ-1 (107 bp)	Fed: na Unfed: na Feral cat 1: na Feral cat 2: na
SARS-CoV-2, E-gene	E_Sarbeco_F1: ACAGGTACGTTAATAGTTAATAGCGT E_Sarbeco_R2: ATATTGCAGCAGTACGCACACA E_Sarbeco_P1: ACACTAGCCATCCTTACTGCGCTTCG [5′]Fam [3′]BHQ-1 (125 bp)	Fed: na Unfed: na Feral cat 1: na Feral cat 2: na

*Notes*: RNA samples were extracted using the AllPrep DNA/RNA/Protein Mini Kit (Qiagen, Valencia, CA, USA) from midguts dissected from laboratory-reared unfed and fed cat fleas [3] and from the pools of fleas collected from feral cats. A SYBR green One-Step real-time RT-PCR assay (BioRad, Hercules, CA, USA) targeting the ORF1b was used for generic detection of coronaviruses [16]. Three SARS-CoV-2-specific RT-PCRs targeting the envelop protein E-coding gene and two targets (IP2 and IP4) of RNA-dependent RNA polymerase gene (RdRp) were conducted using the SuperScript III Platinum One-Step qRT-PCR kit (Thermo Fisher Scientific). The protocols used for RT-PCR are included in the WHO guidelines (https://www.who.int/emergencies/diseases/novel-coronavirus-2019/technical-guidance/laboratory-guidance). The positive controls included a positive sample loaned from the University Hospital of Ciudad Real, Spain and an *in vitro* transcribed RNA derived from the strain BetaCoV_Wuhan_WIV04_2019 (EPI_ISL_402124) loaned by the Pasteur Institute, Paris, France. Real-time RT-PCR was carried out using the CFX96 Touch Real-Time PCR Detection System Thermal Cycler (BioRad, Hercules, CA, USA). Flea samples: Fed and Unfed, laboratory-reared cat flea; Feral cat, pool of feral cat fleas

*Abbreviations*: na, not amplified; Ct, cycle threshold

The analysis identified coronavirus-derived proteins in laboratory-reared cat fleas [3] (Figs. 1 and 2). Identified proteins with proteotypic peptides included coronavirus ORF1ab (protein coverage 9%) encoding for proteins involved in proteolysis, interferon (IFN) antagonism, viral replication and transcription, envelope protein M (coverage 15%), and accessory protein 7b encoded in canonical single guide sgRNA (coverage 19%) [15]. Then, the analysis by real-time RT-PCR of the coronavirus ORF1b [16] resulted in positive signals in laboratory-reared unfed fleas and in fleas collected from one of the feral cats (Fig. 1, samples 2 and 3; Table 1). The three SARS-CoV-2-specific RT-PCRs did not give positive results (Table 1), thus indicating that the identified coronavirus in *C. felis* was not SARS-CoV-2. Our study has limitations. The coronavirus-derived RNA/proteins identified by proteomics and/or RT-PCR in cat flea do not correspond to SARS-CoV-2 because samples from laboratory-reared cat flea were originally processed in 2016 [3], and only two feral cats were included in the study to provide additional support to the presence of coronavirus-like agents in *C. felis*.

The identification of coronavirus-derived RNA in the midgut of an unfed cat flea suggested the possibility of virus infection and replication in fleas. To further explore this possibility, the characterized ACE receptor for SARS-CoV [9] was identified in the exoproteome of *C. felis* with high sequence homology to fruit fly *Drosophila melanogaster* ACE [3]. The analysis showed that ACE are highly conserved proteins at both primary and secondary structure in arthropod vectors, bats and humans (Fig. 3a–d). These results suggested a possible interaction of SARS-CoV with cat flea ACE, a necessary step for coronavirus infection of host cells [9].

**Fig. 3:**
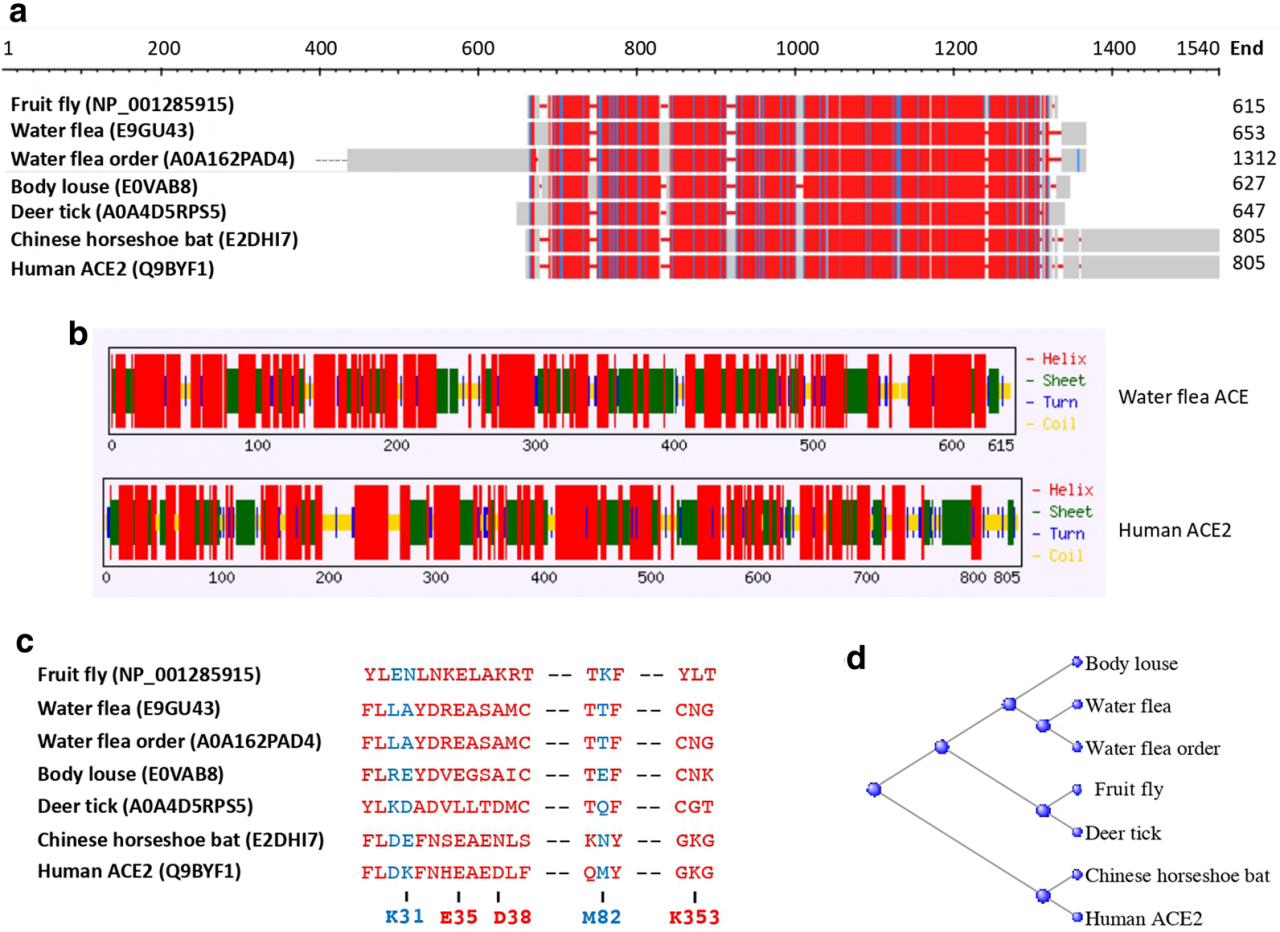
Evolutionary conservation of SARS-CoV receptor ACE protein. **a** Amino acid sequence alignment (Blast E-value = 0.003, max cluster distance = 0.4) was performed with COBALT (https://www.ncbi.nlm.nih.gov/tools/cobalt/cobalt.cgi?LINK_LOC=BlastHomeLink) using protein sequences for ACE in fruit fly (*Drosophila melanogaster*; Uniprot ID: NP_001285915), water flea (*Daphnia pulex*; E9GU43), water flea order (*D. pulex*; A0A162PAD4), body louse (*Pediculus humanus corporis*; E0VAB8), deer tick (*Ixodes scapularis*; A0A4D5RPS5), Chinese horseshoe bat (*Rhinolophus sinicus*; E2DHI7) and human ACE2 (*Homo sapiens*; Q9BYF1). Conserved regions are highlighted in red. **b** Prediction of fruit fly ACE and human ACE2 proteins secondary structure using CFSSP: Chou & Fasman Secondary Structure Prediction Server (http://www.biogem.org/tool/chou-fasman/index.php). **c** Amino acids K31, E35, D38, M82 and K353 identified as involved in the interface between SARS-CoV and human ACE2 [16]. **d** Slanted cladogram of ACE protein sequences using the Neighbor joining algorithm (max seq difference = 0.85, distance = Grishin protein) at NCBI tree viewer (https://www.ncbi.nlm.nih.gov/tools/treeviewer/)

In relation to COVID-19, these findings raise several questions that may be relevant for the identification of the possible SARS-CoV-2 animal hosts and transmission routes. Recent results support that infections in domestic cats likely come from the human-to-pet transmission of the SARS-CoV-2 [17]. Furthermore, current evidence suggests that pets are probably dead-end-hosts with small risk of transmission to humans [10]. Nevertheless, our results suggested that arthropod ectoparasite vectors of cats such as *Ctenocephalides* spp. fleas may act as biological and/or mechanical vectors of SARS-CoV. Although preliminary, these results indicate the possibility of ectoparasites acting as reservoirs and vectors of SARS-CoV and related beta-coronaviruses although with little disease risk due to systemic transmission route, low viremia when compared to vector-borne flaviviruses, virus attenuation or other unknown factors.

Future studies should further explore the presence of SARS-CoV-2 in fleas collected from cats in regions with high COVID-19 prevalence. Although the results support conservation of ACE proteins in arthropod vectors such as fleas and ticks, their functional interaction with SARS-CoV-2 and other SARS-CoV needs to be investigated. Therefore, experimental infestation of SARS-CoV-2-infected cats with cat fleas is required to prove the possible transmission of the coronavirus by arthropod vectors.

In conclusion, our results provide insights that may help epidemic surveillance and preventive measures against COVID-19. In addition to described possible routes of SARS-CoV-2 transmission between bats and humans [7], the findings of our study suggest that additional research is required to explore the spread of COVID-19 and other coronavirus-caused diseases *via* intermediate animal hosts and arthropod vectors such as fleas and ticks. Nevertheless, based on this preliminary evidence, we recommend preventing flea infestations in cats and reducing human exposure to cat fleas as preventive measures for COVID-19.

## Data Availability

All data generated or analysed during this study are included in this published article.
